# Protein Tyrosine Phosphatase 1B (PTP1B): A Potential Target for Alzheimer’s Therapy?

**DOI:** 10.3389/fnagi.2017.00007

**Published:** 2017-01-31

**Authors:** Marcelo N. N. Vieira, Natalia M. Lyra e Silva, Sergio T. Ferreira, Fernanda G. De Felice

**Affiliations:** ^1^Institute of Medical Biochemistry Leopoldo de Meis, Federal University of Rio de JaneiroRio de Janeiro, Brazil; ^2^Institute of Biophysics Carlos Chagas Filho, Federal University of Rio de JaneiroRio de Janeiro, Brazil; ^3^Centre for Neuroscience Studies, Department of Biomedical and Molecular Sciences, Queen’s UniversityKingston, ON, Canada

**Keywords:** Alzheimer’s disease, protein tyrosine phosphatase 1B, diabetes, synaptic plasticity, neuroinflammation, insulin signaling, leptin signaling, endoplasmic reticulum stress

## Abstract

Despite significant advances in current understanding of mechanisms of pathogenesis in Alzheimer’s disease (AD), attempts at drug development based on those discoveries have failed to translate into effective, disease-modifying therapies. AD is a complex and multifactorial disease comprising a range of aberrant cellular/molecular processes taking part in different cell types and brain regions. As a consequence, therapeutics for AD should be able to block or compensate multiple abnormal pathological events. Here, we examine recent evidence that inhibition of protein tyrosine phosphatase 1B (PTP1B) may represent a promising strategy to combat a variety of AD-related detrimental processes. Besides its well described role as a negative regulator of insulin and leptin signaling, PTB1B recently emerged as a modulator of various other processes in the central nervous system (CNS) that are also implicated in AD. These include signaling pathways germane to learning and memory, regulation of synapse dynamics, endoplasmic reticulum (ER) stress and microglia-mediated neuroinflammation. We propose that PTP1B inhibition may represent an attractive and yet unexplored therapeutic approach to correct aberrant signaling pathways linked to AD.

## Introduction

There are currently no disease-modifying therapies for Alzheimer’s disease (AD), and treatments offer limited, temporary improvement in quality of life (Rafii and Aisen, 2015). This scenario drives scientists and pharmaceutical companies into an intense search for effective therapies for AD. Unfortunately, most if not all therapeutics that showed promise in preclinical models failed to translate into effective therapies, as evidenced by numerous unsuccessful clinical trials. Because AD comprises a broad range of deregulated processes taking place concomitantly, drugs acting on multiple aberrant processes hold promise as candidates for AD therapeutics. Herein, we discuss recent evidence indicating that protein tyrosine phosphatase 1B (PTP1B) inhibitors fulfill this criterion.

A pivotal event in AD pathogenesis is the buildup in the brain of amyloid-β oligomers (AβOs), neurotoxins that trigger synapse failure and lead to cognitive impairment (Ferreira and Klein, 2011; Ferreira et al., 2015; Selkoe and Hardy, 2016). In neurons, AβOs attack synapses (Lacor et al., 2004), altering membrane receptor composition (Lacor et al., 2007; De Felice et al., 2009; Jürgensen et al., 2011), impairing synaptic plasticity (Lambert et al., 1998; Walsh et al., 2002), and ultimately leading to synapse loss. Several signaling pathways germane to learning and memory are affected by AβOs. Some of those are initiated by receptor tyrosine-kinases (RTKs) such as the insulin receptor (IR; De Felice et al., 2009; Ma et al., 2009), the leptin receptor (LepR; Marwarha et al., 2011; Maioli et al., 2015) and the brain-derived neurotrophic factor (BDNF) receptor, TrkB (Tong et al., 2004; Echeverria et al., 2007). Additionally, AβOs activate microglia, triggering exacerbated release of proinflammatory cytokines implicated in memory impairment and mood alterations in mouse models of AD (Ledo et al., 2013, 2016).

PTP1B emerged recently as a regulator of a variety of processes within the central nervous system (CNS), many of which therapeutically relevant for AD. Increased PTP1B activity is associated with defective neuronal insulin and leptin signaling (Zabolotny et al., 2002; Pandey et al., 2013, 2014), pathways that are impaired in AD (Bomfim et al., 2012; Bonda et al., 2014). Significantly, down-regulation of PTP1B restores hypothalamic insulin and leptin signaling (Chiarreotto-Ropelle et al., 2013; Lindtner et al., 2013; Pandey et al., 2013, 2014; Yu et al., 2013). PTP1B down-regulates neuronal BDNF-TrkB pathway, whereas PTP1B inhibition boosts BDNF signaling (Ozek et al., 2014; Krishnan et al., 2015). Importantly, mice lacking PTP1B in the hippocampus and cortex displayed improved performance in the Barnes maze (Fuentes et al., 2012), posing this phosphatase as a negative regulator of spatial memory. PTP1B negatively regulates hippocampal store-operated calcium entry (nSOC; Koss et al., 2013), an essential process for the stabilization of mushroom spines that is impaired in transgenic AD mice (Sun et al., 2014; Zhang H. et al., 2015). Furthermore, PTP1B is up-regulated by endoplasmic reticulum (ER) stress (Agouni et al., 2011; Popov, 2012; Hakim et al., 2015), a neuronal response activated by AβOs and implicated in synapse loss and cognitive decline in AD (Kam et al., 2013; Lourenco et al., 2013). Finally, PTP1B is highly expressed in hippocampal microglia (Pei et al., 1994), and was recently described as a positive regulator of neuroinflammation (Song et al., 2016).

In the following sections, we review evidence suggesting that PTP1B modulates several CNS processes relevant to the physiopathology of AD, making it an attractive target to be explored in AD pharmacotherapy. Inhibiting PTP1B appears as a promising, yet neglected strategy to combat multiple aspects of AD.

## Insulin Signaling

**Figure 1:**
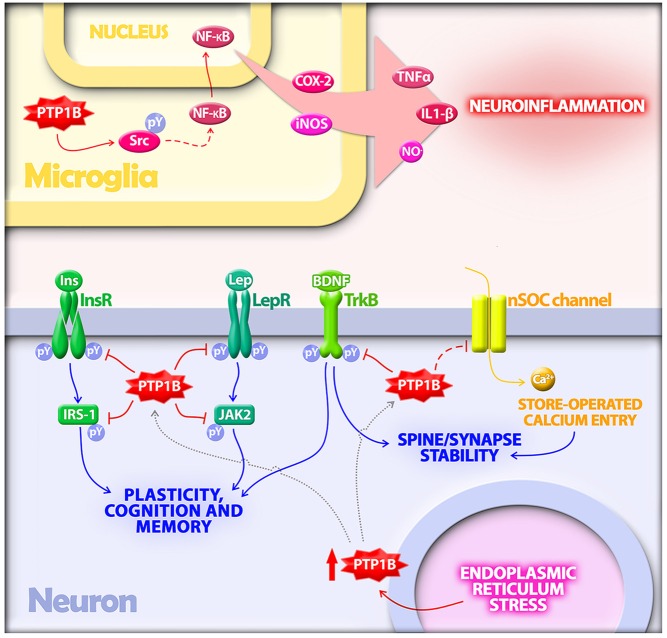
**Protein tyrosine phosphatase 1B (PTP1B) regulates multiple mechanisms implicated in the pathogenesis of Alzheimer’s disease (AD).** In microglia, PTP1B is a positive regulator of neuroinflammation. PTP1B activates Src via dephosphorylation at a negative regulatory site. Src, in turn, indirectly activates NF-κB, a transcriptional regulator of proinflammatory mediators including TNF-α, IL-1β, COX-2 and inducible nitric oxide synthase (iNOS). In neurons, PTP1B is upregulated by endoplasmic reticulum (ER) stress, a cellular response activated by amyloid-β oligomers (AβOs) in AD. Elevated PTP1B inhibits signaling by receptor tyrosine kinases germane to synaptic plasticity, cognition and memory. Substrates for PTP1B in neurons that have been implicated in AD include the insulin receptor (InsR) and its substrate IRS-1, the leptin receptor (LepR) and its immediate downstream effector Janus kinase 2 (JAK2), and the brain-derived neurotrophic factor (BDNF) receptor (TrkB). PTP1B further regulates neuronal store-operated calcium entry (nSOC), a mechanism required for spine/synaptic stability found to be impaired in models of AD.

Insulin signaling is initiated by activation of IR autophosphorylation at tyrosine residues upon insulin binding (Guo, 2014). The immediate effectors IRS-1 and IRS-2 (IR substrate 1 and 2) are then recruited and activated by tyrosine phosphorylation to propagate intracellular signaling (Guo, 2014). PTP1B dephosphorylates tyrosine residues in IR and IRS-1 (Figure 1), reducing insulin sensitivity and shutting down signaling (Goldstein et al., 2000; Bakke and Haj, 2015). PTP1B deficient mice are hypersensitive to insulin and present low basal glycemia and insulinemia (Elchebly et al., 1999), and inhibiting PTP1B improves insulin signaling and reverses T2D phenotypes (Malamas et al., 2000; Zinker et al., 2002; Gum et al., 2003; Tamrakar et al., 2014). Conversely, exacerbated PTP1B activity underlies insulin resistance in T2D (Zabolotny et al., 2002; González-Rodríguez et al., 2010).

An epidemiological correlation between AD and T2D exists, with each disease increasing the risk of developing the other (Ott et al., 1996; Craft and Watson, 2004; De Felice, 2013a,b). However, the mechanisms underlying this connection remain elusive. A breakthrough discovery that contributed to current understanding of such mechanisms was that neurons exposed to AβOs become insensitive to insulin (Zhao et al., 2008; De Felice et al., 2009). Further studies showed that AβOs impair insulin signaling by increasing IRS-1 inhibitory serine phosphorylation and decreasing activating tyrosine phosphorylation (Bomfim et al., 2012). Importantly, defective insulin signaling was confirmed in *post mortem* AD brains (Bomfim et al., 2012; Talbot et al., 2012). Conversely, boosting insulin signaling protects synapses against AβOs toxicity (De Felice et al., 2009). These discoveries paved the way for a whole new aspect of AD, which has provided important advances of therapeutic relevance. For instance, anti-diabetic drugs developed to treat insulin resistance in T2D have shown promising preclinical results, protecting synapses, preventing inhibition of IRS-1 and, most importantly, ameliorating cognitive phenotypes in animal models of AD (McClean et al., 2011; Bomfim et al., 2012; Hansen et al., 2015; Qi et al., 2016). Those studies have provided molecular grounds for on-going clinical trials aimed at testing the efficacy of intranasal insulin and glucagon-like peptide 1 (GLP-1) analogs in AD (De Felice and Ferreira, 2014).

Thus, it seems reasonable to predict that PTP1B inhibitors—which restore insulin sensitivity in T2D models (Malamas et al., 2000; Zinker et al., 2002; Gum et al., 2003; Panzhinskiy et al., 2013; Tamrakar et al., 2014)—may rescue neurons from defective insulin signaling in AD. Although this hypothesis has not yet been tested directly, there is evidence from non-AD models of neuronal insulin resistance validating PTP1B inhibition as an effective approach to rescue neuronal insulin signaling (Krishnan et al., 2015; Qin et al., 2015a; Zhang Z. Y. et al., 2015).

## Leptin Signaling

In obesity, defective hypothalamic leptin signaling impairs sensing and processing of satiety signals, leading to increased caloric intake and decreased energy expenditure (Halaas et al., 1995; Farooqi et al., 1999; Morton et al., 2006). Ob/Ob mice, which do not produce leptin, exhibit increased food intake and become profoundly obese (Zhang et al., 1994). Leptin signaling is initiated by binding of leptin to LepR, leading to tyrosine autophosphorylation of LepR and subsequent phosphorylation of Janus kinase 2 (JAK2), which propagates downstream intracellular signaling (Iida et al., 1996; Fei et al., 1997).

Strong evidence implicates PTP1B in obesity-associated hypothalamic leptin resistance (Cheng et al., 2002; Zabolotny et al., 2002). PTP1B dephosphorylates LepR and JAK2, functioning as a negative regulator of leptin signaling (Figure 1). PTP1B-null mice are resistant to weight gain induced by high-fat diet (HFD) or by deletion of the leptin gene, suggesting PTP1B inhibition as a strategy to rescue leptin signaling in food intake disorders and obesity (Elchebly et al., 1999; Cheng et al., 2002).

Beyond hypothalamic signaling, leptin plays important roles in the CNS. LepRs are highly expressed in the hippocampus (Huang et al., 1996; Mercer et al., 1996; Scott et al., 2009) where leptin signaling is important for cognition and memory (Irving and Harvey, 2014). Aβ down-regulates hippocampal leptin and LepR expression (Marwarha et al., 2010; Bonda et al., 2014). Interestingly, leptin prevents hippocampal synaptic disruption and neuronal death induced by Aβ (Doherty et al., 2013). Leptin also modifies Aβ levels (Fewlass et al., 2004) and reduces tau phosphorylation in neuronal cells (Greco et al., 2008, 2009a,b). Importantly, neuronal leptin resistance has been described in the AD hippocampus (Bonda et al., 2014; Maioli et al., 2015), further underlining the relevance of defective leptin signaling in AD.

Leptin signaling has been proposed as a neuroprotective target in AD (Gomes et al., 2014; Johnston et al., 2014). Because direct administration of leptin or LepR agonists in conditions of leptin resistance may not result in the desired biological effect, a more attractive approach to boost leptin signaling in AD would be to reverse neuronal leptin resistance. The evidence described above suggests that recovery of leptin sensitivity could be achieved by PTP1B inhibitors.

## Endoplasmic Reticulum Stress

ER stress and activation of the unfolded protein response (UPR) are important toxic mechanisms in AD (Lourenco et al., 2015). We recently demonstrated that AβOs trigger ER stress in hippocampal neurons in a mechanism that requires TNF-α receptor activation (Lourenco et al., 2013). ER stress triggered by TNF-α has also been linked to peripheral insulin resistance in obesity and diabetes (reviewed in Hotamisligil, 2010).

PTP1B localizes predominantly to the cytoplasmic surface of the ER (Haj et al., 2002) and mediates ER stress signaling (Wang et al., 2009). Downregulation of PTP1B ameliorates ER stress in obesity and diabetes models (Delibegovic et al., 2009; Agouni et al., 2011; Owen et al., 2013). Two of the UPR branches activated upon ER stress involve inositol-requiring enzyme 1 (IRE-1) and activating transcription factor 6 (ATF6). Interestingly, PTP1B potentiates IRE-1-mediated ER stress response and its expression is regulated by ATF6 (Gu et al., 2004; Wang et al., 2009). Moreover, recent evidence links hypothalamic ER stress and activation of the UPR to development of PTP1B-mediated leptin resistance and increased food intake following chronic sleep fragmentation in mice (Hakim et al., 2015).

Collectively, data suggest that PTP1B mediates the toxic consequences of neuronal ER stress (Figure 1). It has been hypothesized that PTP1B may be a key link between insulin signaling and ER stress (Popov, 2012). This raises the possibility that PTP1B inhibitors may be able to compensate the detrimental impact of ER stress on synapse stability and cognition in AD.

## Synaptic Plasticity, Stability and Memory

The functions of PTP1B in neurophysiology are gaining traction as novel roles for PTP1B in the brain are discovered. PTP1B has been implicated in a variety of neuronal processes, including some related to synapse biology that are potentially relevant to the pathogenesis of AD.

BDNF is a major regulator of synaptic plasticity. BDNF signaling through its receptor, TrkB, modulates synapse structure and function to produce long-term potentiation (LTP), a form of activity-dependent synaptic plasticity thought to underlie learning and memory (Leal et al., 2015). AD brains display reduced BDNF levels in clinical (Phillips et al., 1991; Connor et al., 1997; Soontornniyomkij et al., 1999) and preclinical disease stages (Peng et al., 2005). Conversely, BDNF is neuroprotective in animal models of AD (Arancibia et al., 2008; Nagahara et al., 2009). Therefore, stimulating BDNF signaling represents an attractive approach in AD therapy.

Recent studies showed that PTP1B downregulates BDNF signaling through dephosphorylation of TrkB (Ozek et al., 2014; Krishnan et al., 2015; Figure 1). *Ptpn1*^−/−^ mice are hypersensitive to BDNF, and pharmacological inhibition of PTP1B increases neuronal responsiveness BDNF (Ozek et al., 2014). PTP1B inhibition may, thus, represent a means to enhance BDNF signaling, improving synaptic plasticity and cognition in AD.

PTP1B has further been implicated in hippocampal synapse formation and learning (Fuentes et al., 2012). PTP1B is present in dendritic spines of hippocampal neurons, and functional or genetic PTP1B deficiency affects spine morphology and leads to disorganization of pre- and post-synaptic terminals. Interestingly, mice lacking PTP1B in the hippocampus and cortex exhibit improved performance in the Barnes maze compared to wild-type controls, posing this phosphatase as a negative regulator of spatial memory (Fuentes et al., 2012).

Neuronal store-operated calcium entry (nSOC) is essential for stabilization of mushroom spines in hippocampal neurons (Sun et al., 2014). AD mice exhibit impaired nSOC, which causes destabilization and loss of spines through a mechanism involving overactivation of metabotropic glutamate receptor 5 (mGluR5; Sun et al., 2014; Zhang H. et al., 2015). PTP1B is a negative regulator of hippocampal nSOC (Koss et al., 2013; Figure 1), suggesting PTP1B inhibition may restore deficient nSOC.

Collectively, these findings suggest PTP1B inhibition may result in improved synapse plasticity, function and stability, ultimately enhancing cognitive performance.

## Microglia-Mediated Neuroinflammation

Chronic neuroinflammation is an important feature of AD (Heneka et al., 2015; Heppner et al., 2015). Evidence for activated microglia has been described in transgenic AD mice (Frautschy et al., 1998; Bornemann et al., 2001) and in AD brains (Cagnin et al., 2001; Edison et al., 2008). Microglia are implicated in cognitive impairment in AD through sustained secretion of neurotoxic cytokines (Wang et al., 2015) and synapse pruning (Hong et al., 2016; Lui et al., 2016). Injection of AβOs in mouse brains induces microglia-mediated neuroinflammation (Xu et al., 2016), leading to memory impairment and mood alterations (Ledo et al., 2013, 2016). Direct activation of microglia by AβOs was recently demonstrated in primary microglial cultures (Ledo et al., 2016).

Activated microglia are the main source of proinflammatory cytokines such as TNF-α and IL-1β in the brain (Wang et al., 2015). TNF-α is implicated in memory impairment caused by AβOs (Bomfim et al., 2012; Lourenco et al., 2013). Activation of TNF-α signaling is associated with inhibition of IRS-1 in hippocampal neurons (Bomfim et al., 2012). TNF-α is also implicated in peripheral insulin resistance in diabetes (Hotamisligil et al., 1993; Hotamisligil and Spiegelman, 1994), and it has been proposed that neuronal insulin resistance induced by TNF-α may underlie the connection between diabetes and AD (De Felice and Ferreira, 2014). IL-1β has been described as a mediator of cognitive impairment associated with peripheral and central inflammation by disrupting hippocampal synaptic plasticity (Di Filippo et al., 2013; Erion et al., 2014). These lines of evidence support dampening microglia-mediated neuroinflammation as an attractive therapeutic approach in AD (Ransohoff, 2016; Santos et al., 2016; Wes et al., 2016).

PTP1B is regulated by proinflammatory signals and is highly expressed in hippocampal microglia in AD (Pei et al., 1994). Interestingly, a recent study unraveled a novel role for PTP1B as a positive regulator of neuroinflammation (Song et al., 2016). PTP1B levels are increased in LPS injected brain, and PTP1B overexpression potentiates microglial responses via dephosphorylation/activation of Src and nuclear translocation of NF-κB, leading to increased expression of proinflammatory molecules including TNF-α, IL-1β, cyclooxigenase-2 and inducible nitric oxide synthase (iNOS; Figure 1). Significantly, LPS-induced neuroinflammation is attenuated by pharmacological PTP1B inhibitors (Song et al., 2016). Conversely, TNF-α increases PTP1B expression via NFκB in adipose tissue of HFD mice (Zabolotny et al., 2008) and in organotypic hypothalamic cultures (Ito et al., 2012), further exacerbating inflammation in a feed-forward mechanism. This suggests that pharmacological inhibition of PTP1B may constitute a therapeutic strategy to counteract neuroinflammation.

## PTP1B Inhibitors as Drug Candidates for Neurological Diseases

PTP1B has long been pursued as a therapeutic target in human diseases, particularly in diabetes and obesity (Zhang and Lee, 2003). Multiple PTP1B inhibitors have been developed and tested in preclinical models, validating the concept of PTP1B inhibition as an effective therapeutic approach for diabetes (Tamrakar et al., 2014). Nevertheless, certain structural features of PTP1B complicate the development of small molecule inhibitors with fundamental characteristics required for drug candidates, namely, specificity/selectivity and bioavailabilty. First, the active sites of protein tyrosine phosphatases (PTPs) are highly conserved among the more than 100 family members (Tonks, 2013). Therefore, inhibitors designed to bind to the active site of PTP1B often inhibit other PTPs as well, leading to off-target effects (Tamrakar et al., 2014; Tautz, 2015). Second, most inhibitors developed so far are phosphotyrosine-mimicking molecules bearing a charged group, which drastically affects pharmacokinetics (Tautz, 2015). For those reasons, few PTP1B inhibitors have reached clinical trials, and none have made it through phase II tests (Tamrakar et al., 2014; Tautz, 2015).

In AD, the blood-brain barrier (BBB) represents an additional obstacle drugs need to overcome to reach the CNS. Fortunately, however, significant advances have been achieved in developing effective PTP1B inhibitors with potential for clinical use. For example, an important recent study provided compelling evidence that potent and selective PTP1B inhibitors administered peripherally (e.g., intraperitoneally or subcutaneously) inhibit PTP1B activity in the brain (Krishnan et al., 2015). *MeCP2* deficient mice, a model of Rett syndrome, exhibit increased PTP1B expression, leading to defective brain insulin signaling and impaired glucose metabolism. This was associated with neurological phenotypes in female mice and reduced lifespan in male mice, recapitulating Rett syndrome in humans. Remarkably, long-term systemic treatment with two distinct PTP1B inhibitors, CPT157633 and UA0713, rescued disease phenotypes in* MeCP2* deficient mice. Moreover, PTP1B inhibitors increased tyrosine phosphorylation of TrkB, leading to enhanced signaling in response to BDNF in the forebrains of *MeCP2* deficient mice (Krishnan et al., 2015).

Another recent study showed that intraperitoneal administration of trodusquemine, a selective BBB-permeant (Ahima et al., 2002; Lantz et al., 2010) PTP1B inhibitor, relieved anxiety in *LMO4* knockout mice exhibiting impaired endocannabinoid signaling due to increased PTP1B activity in amygdala (Qin et al., 2015b). Those studies support the feasibility of using peripherally administered PTP1B inhibitors to treat CNS disorders.

## Conclusion

Compelling recent findings suggest PTP1B holds potential as a therapeutic target in AD. Results indicate that PTP1B regulates distinct CNS responses depending on cell type: while PTP1B modulates insulin and leptin signaling in neurons, it regulates astrocyte differentiation (Yamada et al., 2013) and proinflammatory responses in microglia. Because PTP1B participates in several cellular/molecular processes linked to AD pathogenesis (Figure 1), compounds capable of reaching the CNS and inhibiting PTP1B activity in neurons and/or glial cells may rescue multiple aberrant processes associated with cognitive decline and neurodegeneration in AD.

Detailed preclinical studies are warranted to validate the potential benefits of PTP1B inhibition in AD models. Because PTP1B may interfere with pathological mechanisms that operate at different disease stages, it will be important to investigate whether PTP1B inhibition delays, prevents or slows down disease progression or temporarily ameliorates symptoms.

Finally, the above described pathological mechanisms in which PTP1B has been implicated are not exclusively associated with AD, but also with other neurological disorders. This raises the possibility that PTP1B inhibition may be useful for treatment of other brain disorders related to metabolic deregulation, and perhaps even in normal, age-related cognitive decline.

## Author Contributions

MNNV: development of the subject matter, drafting of the article, conception and design of the figure, critical revision of the article, final approval of the version to be published; NMLS and FGF: development of the subject matter, drafting of the article, critical revision of the article, final approval of the version to be published; STF: discussion of contents, critical revision of the article, final approval of the version to be published.

## Funding

Work in the authors’ laboratory is funded by grants from Conselho Nacional de Desenvolvimento Científico e Tecnológico (CNPq), Fundação de Amparo à Pesquisa do Estado do Rio de Janeiro (FAPERJ), National Institute for Translational Neuroscience (to FGF and STF) and Canadian Institutes of Health Research (CIHR; to FGF). MNNV is a former postdoctoral fellow of CNPq and a current fellow of FAPERJ. Studies on PTP1B inhibition as a therapy to Alzheimer’s disesase are funded by the International Society for Neurochemistry (ISN) Committee for Aid and Education in Neurosciences (CAEN) Career Interruption, Re-entry Grant (to MNNV). NMLS is a pre-doctoral fellow of CNPq.

## Conflict of Interest Statement

The authors declare that the research was conducted in the absence of any commercial or financial relationships that could be construed as a potential conflict of interest.
